# Protocol for a systematic review and meta-analysis of studies on the use of brain natriuretic peptide and N-terminal brain natriuretic peptide levels in the diagnosis of cardiopulmonary edema in acute respiratory failure

**DOI:** 10.1186/s13643-021-01869-1

**Published:** 2021-12-16

**Authors:** Takero Terayama, Takuya Taniguchi, Ryosuke Imai, Keisuke Anan, Takuo Yoshida, Koichi Ando, Satoshi Okamori, Yohei Okada

**Affiliations:** 1 Department of Psychiatry, School of Medicine, National Defense Medical College, Saitama, Japan; 2 Department of Cardiovascular Medicine, Otsu City Hospital, Otsu, Shiga Japan; 3 Department of Pulmonary Medicine, Thoracic Center, St. Luke’s International Hospital, Tokyo, Japan; 4 Department of Healthcare Epidemiology, School of Public Health in the Graduate School of Medicine, Kyoto University, Kyoto, Japan; 5 Intensive Care Unit, Department of Anesthesiology, Jikei University School of Medicine, Nishi-Shinbashi Minato-ku, Tokyo, Japan; 6 Division of Allergology and Respiratory Medicine, Department of Medicine, Showa University School of Medicine, 1-5-8 Hatanodai, Shinagawa-ku, Tokyo, Japan; 7 Division of Pulmonary Medicine, Department of Medicine, Keio University School of Medicine, Shinjuku-ku, Tokyo, Japan; 8 Department of Primary Care and Emergency Medicine, Graduate School of Medicine, Kyoto University, Preventive Services, School of Public Health, Kyoto, Japan

**Keywords:** Respiratory failure, Acute respiratory distress syndrome, Cardiopulmonary edema, Brain natriuretic peptide, Meta-analysis, Diagnostic accuracy test

## Abstract

**Background:**

Dyspnea with bilateral pulmonary edema is common among patients in emergency departments (EDs) or intensive care units (ICUs). For the initial management of patients with this condition, cardiopulmonary edema (CPE) must be differentiated from acute respiratory distress syndrome (ARDS) in clinical settings. Brain natriuretic peptide (BNP) and N-terminal brain natriuretic peptide (NT-proBNP) are useful in distinguishing these conditions. However, current data about the use of these indexes are limited. Hence, we planned to perform a systematic review and meta-analysis to determine the accuracy of the two indexes for the diagnosis of CPE.

**Methods:**

We designed and registered a study protocol for a systematic review and meta-analysis. This study aims to determine the diagnostic accuracy of BNP and NT-proBNP based on the standards of the methodology of the Cochrane Handbook for Systematic Reviews of Diagnostic Test Accuracy and the Preferred Reporting Items for a Systematic Review and Meta-analysis of Diagnostic Test Accuracy Studies in reporting the findings of this review. We will search PubMed (MEDLINE), Cochrane Library, Embase, www.ClinicalTrials.gov, International Clinical Trials Registry Platform, and Google Scholar. Randomized controlled trials, cross-sectional studies, and observational cohort studies reporting the accuracy in diagnosing CPE among adult patients with dyspnea and bilateral pulmonary edema will be included in the analysis. There will be no limits regarding language and publication date for this review. Two reviewers will independently screen articles, extract data, evaluate for quality and bias using the Quality Assessment of Diagnostic Accuracy Studies (QUADAS-2), and use Grading of Recommendations Assessment, Development and Evaluation to summarize the strength of body of evidence. Then, a meta-analysis will be performed, and different statistical methods will be used to investigate heterogeneity among studies. A subgroup analysis of elderly patients with left ventricular dysfunction or chronic renal dysfunction will be performed. In the meta-analysis, a hierarchical summary receiver operating characteristic model or a bivariate model will be used in each index test, as appropriate.

**Discussion:**

A systematic review and meta-analysis of the accuracy of BNP and NT-proBNP for the diagnosis of CPE will be conducted. The result of this study can help clinicians to identify an appropriate initial treatment for patients with acute respiratory failure, including those with ARDS and CPE. To the best of our knowledge, this will be the first comprehensive systematic review focusing on ARDS management in a specific population.

**Systematic review registration:**

PROSPERO ID CRD42020201576

**Supplementary Information:**

The online version contains supplementary material available at 10.1186/s13643-021-01869-1.

## Background

Acute respiratory distress syndrome (ARDS) is a type of respiratory failure characterized by the acute onset of bilateral alveolar opacities and hypoxemia diagnosed based on the Berlin Definition [1]. Generally, it has a high mortality and morbidity. Moreover, it affects approximately 200,000 individuals and results in 74,500 deaths annually in the USA [2]. Thus, several intensive managements, such as tracheal intubation, mechanical ventilation, and extracorporeal oxygenation, are required. ARDS is believed to be a secondary insult to the lungs, and it is associated with other primary conditions, such as trauma, burn, and infection. Therefore, in addition to the intensive management mentioned above, diagnosis of the primary condition and appropriate treatment are essential to save the lives of individuals with this condition. However, the diagnosis can be challenging in the early stage of illness [3], particularly among patients with advanced age, multiple comorbidities, and polypharmacy [4, 5].

The Berlin Definition is based on a specified acute time frame (within 7 days from onset or deterioration), presence of bilateral opacities on chest radiography or computed tomography (CT) scan, cause of pulmonary edema that cannot be explained by heart failure or volume overload alone, and hypoxia (PaO_2_/fraction of inspired oxygen [FiO_2_] [P/F] ratio < 300) [1]. The criteria do not include pulmonary artery wedge pressure (PAWP), which is measured using a right atrial catheter, because it is invasive and costly and has low accuracy in clinical estimation [6]. Then, alternative clinical tools for differential diagnosis, such as biomarker levels, alveolar protein concentration, and echocardiogram results, have been explored [3, 7–10]. Some studies reported that brain natriuretic peptide (BNP) and N-terminal brain natriuretic peptide (NT-proBNP) were useful and highly accurate in distinguishing cardiopulmonary edema (CPE) from acute lung injury (ALI)/ARDS [11, 12]. Komiya conducted a systematic review on systemic biomarkers (BNP, NT-proBNP, C-reactive protein, plasma soluble suppression of tumorigenicity-2, heparin-binding protein, and copeptin levels), lung biomarkers (fluid-to-plasma protein ratio and surfactant apoprotein-A concentration in the bronchoalveolar lavage fluid), and imaging studies (chest ultrasonography, chest CT scan )[13]. This study showed that BNP and NT-proBNP were the most commonly used systemic biomarkers. Moreover, BNP and NT-proBNP are extremely simple to use as they are available in any clinical setting. However, other methods may not be available particularly in low-resource settings.

Current data on whether BNP and NT-proBNP are beneficial for differential diagnosis are limited. Martindale and colleagues conducted a systematic review and meta-analysis on the diagnosis of acute heart failure using BNP and NT-proBNP among patients with dyspnea in an emergency department (ED) setting. They pooled patient-level BNP data from six studies and NT-proBNP data from five studies. Results showed that the areas under the receiver operating characteristic (ROC) curve were 0.86 (95% confidence interval [CI] = 0.83–0.86) for BNP and 0.76 (95% CI = 0.74–0.78) for NT-proBNP [14]. Although their review is helpful for physicians treating heart failure, it would be inadequate to be adopted for the practice of ARDS due to the wide range of patients involved. ARDS is a serious condition that is treated in intensive care units, so it is necessary to conduct a systematic review in a more appropriate and specific population. On the other hand, Komiya et al. conducted a systematic review including BNP and NT-proBNP of patients that is in line with our objective, but they did not quantitative synthesis, and so their review is also inadequate for direct adoption to ARDS practice [13].

Hence, we planned to conduct a systematic review and meta-analysis on the diagnostic accuracy of both plasma BNP and NT-proBNP among adult patients with acute respiratory failure in ED and ICU settings based on rigorous methodological guidelines [15, 16].

## Methods/design

This is a protocol for a systematic review and meta-analysis on the diagnostic test accuracy (DTA) of BNP and NT-proBNP for the detection of CPE in patients with acute respiratory failure. We will adhere to the standards of the methodology of the Cochrane Handbook for Systematic Reviews of DTA [17] and the Preferred Reporting Items for Systematic Reviews and Meta-analyses of Diagnostic Test Accuracy Studies [16] in reporting the findings of this review.

### Objectives

#### Primary objective

To determine the accuracy of BNP and NT-proBNP for the diagnosis of CPE in patients with acute respiratory failure in ED or ICU.

### Criteria for studies included in this review

#### Types of studies

We will include all reports on the accuracy of plasma BNP or NT-proBNP for the diagnosis of CPE among adult patients with acute respiratory failure. Moreover, the study will comprise prospective or retrospective observational (cohort or cross-sectional) studies or secondary analysis of randomized controlled trials. However, those without sufficient diagnostic test accuracy data, namely true-positive (TP), false-positive (FP), true-negative (TN), and false-negative (FN) values, based on the reference standard will be excluded.

#### Participants

The target participants are as follows:

1. Adult patients aged 15 years or older.
2. Patients with acute respiratory failure, dyspnea, and hypoxia who were admitted in the ED or ICU.
3. Patients with bilateral pulmonary edema on imaging studies, such as radiography and CT scan.

The summary of inclusion criteria for this review is demonstrated in Table 1.

**Table 1:** Inclusion criteria

Study characteristics	Inclusion criteria
Population	Adult > 15 years of age with acute respiratory failure, dyspnea, and hypoxia who were admitted in the ED or ICU
Index tests	BNP and NT-pro BNP
Reference test	Final diagnosis made by experts, such as cardiologists and emergency physicians
Outcomes	True and false positives, true and false negatives
Study designs	All prospective, retrospective, or randomized controlled trials except for case-control studies and case series.
Language	No limits
Publication date	No limits
Publication status	Including unpublished studies

*ED* emergency department, *ICU* intensive care unit, *BNP* brain natriuretic peptide, *NT-proBNP* N-terminal brain natriuretic peptide

#### Index test

The index tests are plasma BNP and NT-proBNP assays using any type of method. We will report these index tests as positive or negative based on the study threshold cutoffs. Studies evaluating both BNP and NT-proBNP in a similar study population will also be included.

BNP and NT-proBNP are different indexes widely used to diagnose heart failure [18, 19]. Currently, an alternative test for cardiac biomarkers is not available. To distinguish ARDS from CPE in patients with acute respiratory failure and bilateral pulmonary edema, the BNP and NT-proBNP tests can be used in addition to echocardiogram, chest radiography, and physical examination.

BNP is synthesized as a prohormone (proBNP), which is then cleaved into the active fragment BNP (32-amino-acid, C-terminal fragment) and the inert fragment NT-proBNP (inactive 76 amino-acid, N-terminal fragment). They are synthesized and released into the circulation by cardiac ventricular myocytes in response to volume expansion and possible increased wall stress. Both are cleared mainly by the kidneys. However, NT-proBNP has a longer half-life (mean: 120 vs. 20 min), and it is more stable than BNP in vitro [20, 21]. The serum BNP and NT-proBNP levels may vary due to kits used in the examination or under some conditions, such as renal dysfunction, obesity, drug-related disorder, inflammation, and cancer [22–25].

#### Reference standard

The reference standard for the final diagnosis made by experts, such as cardiologists and emergency physicians, refers to all available patients’ information, including clinical features and response to treatment.

The difference between CPE and ARDS in ED and ICU settings commonly comprise the combined results for physical examination, echocardiogram, and invasive evaluation (e.g., PAWP). In echocardiogram, ejection fraction (EF), left ventricular end-diastolic dimension, and diameter of the inferior vena cava are often evaluated. However, the inter-observer agreement for the diagnosis can be low unless these examinations are performed by expert sonographers, including cardiologists. Moreover, in recent years, PAWP has been found to provide inaccurate clinical estimation [26], and there is no clear evidence showing its benefits [27, 28]. The trend was identified based on the Berlin Definition, in which low PAWP (<18 mmHg) is no longer required for the assessment of ARDS [1].

#### Target conditions

The target condition is CPE, which should have causes different from those of acute respiratory failure during the initial treatment. Acute respiratory failure with bilateral lung infiltrates on chest radiography or CT scan is common in the ED and ICU settings. In this review, CPE was defined as bilateral lung infiltrates on radiography or CT scan based on the reference standard (18). The timing of the diagnosis ranges from the early stage to the late stage of illness, such as during hospital discharge.

#### Clinical settings

The clinical settings will be in the ED and ICU.

### Search methods used to identify studies

#### Electronic searches

An electronic search strategy has been developed in collaboration with librarians. To identify all prospective, retrospective, or randomized controlled trials, we will search MEDLINE (via PubMed; from 1966 to the present) and Embase, Cochrane Central Register of Controlled Trials in the Cochrane Library. We will search for ongoing and unpublished studies at www.clinicaltrials.gov, the International Clinical Trials Platform (www.who.int/ctrp/en), and Google Scholar. There are no limits regarding language and publication date for this review. We have outlined the search strategy in Additional file 1: Appendix 1. Moreover, the reference lists of relevant articles will be hand searched.

### Data collection and analysis

#### Selection of studies

Two or more authors will independently screen all articles identified using our search strategy based on the inclusion criteria of this review. Screening will be a two-step process (initial title/abstract screening and full-text screening). Disagreements among reviewers will be resolved via a consensus or third-party reviewer. After the full-text screening, a list of excluded studies with reasons will be provided in the Additional file 1: Appendix of the final report.

#### Data extraction and management

Two or more authors will develop the data extraction sheet with the following information:

1. Study characteristics: author, year of publication, country where the study was conducted, design, sample size, clinical settings, and funding source.
2. Population characteristics: inclusion/exclusion criteria, number of dropouts with reason, and demographic characteristics of the participants (such as age and sex).
3. Index test: timing of sampling, method of examination, time to result, and name of the person who conducted the test.

Reference standard: method of examination, time to result, and name of the person who performed the examination.

4) Information regarding quality assessment items based on the Quality Assessment of Diagnostics-Accuracy Studies 2 (QUADAS-2) assessment system [15].
5) Outcomes: Based on the information in the 2 × 2 table, we will assess diagnostic accuracy parameters, such as TP, FP, TN, and FN values.

#### Assessment of methodological quality

Two or more investigators will independently evaluate and report the risk of bias using the QUADAS-2 tool [15]. We will assess four domains for the risk of bias, which are as follows: patient selection, index test, reference test, and flow and timing. Moreover, applicability concerning the first three domains will be evaluated. For each domain, we will respond to the questions with a Yes/No/Unclear answer, and the risk of bias will be considered as Low/High/Unclear.

A statistical assessment of publication bias will not be performed. There is no evidence of publication bias in the systematic reviews of diagnostic accuracy, and the methods used in assessing publication bias are not reliable when applied to diagnostic accuracy studies.

The sources of bias in diagnostic accuracy studies include those related to the patients (spectrum bias and selection bias), index test (information bias), reference test (misclassification bias, partial verification bias, differential verification bias, incorporation bias, disease progression bias, and information bias), and data analysis (excluded data bias).

We will summarize the strength of body of evidence using the systematic review using the “Grading of Recommendations Assessment, Development and Evaluation” approach [29] with classification based on study design and limitations, indirectness, inconsistency, imprecision, and publication bias [30].

#### Statistical analysis and data synthesis

We will individually analyze BNP and NT-proBNP. In the included studies, the reference standard (final diagnosis made by experts) will have dichotomous outcomes, and the index tests will have thresholds at which the diagnostic accuracy parameters will be calculated. For all studies, we will establish 2 × 2 tables (multiple tables for a study with multiple thresholds) with data on TP, FP, FN, and TN values in each study. The diagnostic odds ratio will be also calculated, which is a measure of the discriminative power of a test that has been considered a good indicator of test performance [31, 32]. We will use forest plots with 95% confidence intervals (CIs) to assess the sensitivity and specificity in each study. To visually assess the correlation between both indices, the summary of the ROC curve will be plotted when the studies have different cutoffs or the results were presented as circles in the ROC space when the studies had a similar cutoff for reporting sensitivity versus 1-specificity. We expect that the included studies will use different threshold cutoffs for the assessment of sensitivity and specificity because no consensus has been established as to the optimal threshold cutoff of BNP or NT-proBNP for the diagnosis of CPE. In the meta-analysis, we will use a hierarchical summary receiver operating characteristic model to pool data and to estimate and summarize the receiver operating characteristic curve when the studies use different cutoffs. A bivariate model will also be used when the studies use similar cutoffs.

All analyses will be performed using the STATA, SAS (SAS Institute Inc., Cary, NC, the USA), or Review Manager 5 software (Cochrane Collaboration, London, the UK).

#### Assessment of heterogeneity

Heterogeneity was assessed using the *I*^2^ statistical method, with *I*^2^ > 50% or *p* value  < 0.05 indicating significant heterogeneity. We want to perform subgroup analyses if the following data are available: age (elderly/adult) and past medical history (left ventricular dysfunction or chronic renal insufficiency)

#### Sensitivity analyses

We will assess for robustness by excluding studies with a high risk of bias.

#### Assessment of reporting bias

We will not assess publication or reporting bias as there is no accepted method that can be used for its evaluation in a meta-analysis of diagnostic test accuracy studies [33].

## Discussion

This systematic review and meta-analysis aims to provide a summary of existing knowledge on the accuracy of cardiac biomarkers for the diagnosis of CPE among patients with acute respiratory failure. To the best of our knowledge, this protocol will be the first in this field.

Any modifications made to our protocol during the review will be reported in the final paper. We plan to submit the review in a peer-reviewed journal with articles often read by physicians working in the ICU. Furthermore, we believe that this review will also be interesting to non-experts in ARDS.

This protocol can help physicians in selecting an appropriate initial management for patients with acute respiratory failure and bilateral pulmonary edema.

### Registration

The protocol for our review has been registered with PROSPERO, the international prospective register of systematic review (PROSPERO 2020:CRD42020201576).

## Supplementary Information

**Additional file 1: Appendix 1**. Search strategy for electronic databases.

## Data Availability

Data sharing is not applicable to this article as no datasets were generated or analyzed during the current study. Materials during the current study are available from the corresponding author upon reasonable request.

## Abbreviations

ARDS: Acute respiratory distress syndrome
CT: Computed tomography
FiO_2_: Fraction of inspired oxygen
P/F ratio: PaO_2_/fraction of inspired oxygen ratio
PAWP: Pulmonary artery wedge pressure
BNP: Brain natriuretic peptide
NT-proBNP: N-terminal brain natriuretic peptide
CPE: Cardiopulmonary edema
ALI: Acute lung injury
ED: Emergency department
ROC: Receiver operating characteristic
ICU: Intensive care unit
DTA: Diagnostic test accuracy
TP: True-positive
FP: False-positive
TN: True-negative
FN: False-negative
EF: Ejection fraction
QUADAS-2: Quality Assessment of Diagnostic Accuracy Studies 2
CI: Confidence interval
