# Supplementary material for: Protocol for a systematic review and meta-analysis of studies on the use of brain natriuretic peptide and N-terminal brain natriuretic peptide levels in the diagnosis of cardiopulmonary edema in acute respiratory failure
Source: Syst Rev. 2021 Dec 16;10:314. doi: 10.1186/s13643-021-01869-1 (PMC8675464; doi:10.1186/s13643-021-01869-1)
Supplement: Supplementary file 1 — Additional file 1: Appendix 1. Search strategy for electronic databases. [file 13643_2021_1869_MOESM1_ESM.docx]

**Appendix 1. Search strategy for electronic databases**

**Search strategy for MEDLINE (via PubMed)**

| #1 | Natriuretic Peptide, Brain[mh] OR BNP OR B type natriuretic peptide OR brain natriuretic peptide |
| --- | --- |
| #2 | Respiratory distress syndrome, adult[mh] OR shock lung OR acute respiratory distress syndrome OR Adult respiratory distress syndrome |
| #3 | ARDS OR ALI |
| #4 | Respiratory Insufficiency[mh] OR Respiratory Failure OR Respiratory Depression OR Ventilatory Depression OR Depressions, Ventilatory OR acute respiratory failure |
| #5 | Acute lung injury[mh] OR Acute lung injuries OR lung injury |
| #6 | severe acute respiratory syndrome[mh] OR severe acute respiratory syndrome |
| #7 | Pulmonary edema[mh] OR Cardiogenic pulmonary edema |
| #8 | #2 OR #3 OR #4 OR #5 OR #6 OR #7 |
| #9 | #1 AND #8 |
| #10 | animals[mh] NOT humans[mh] |
| #11 | #9 NOT #10 |

**Search strategy for Cochrane Central Register of Controlled Trials**

| ID | Search |
| --- | --- |
| #1 | MeSH descriptor: [Natriuretic Peptide, Brain] explode all trees |
| #2 | bnp |
| #3 | b type natriuretic peptide |
| #4 | natriuretic peptide, brain |
| #5 | #1 OR #2 OR #3 OR #4 |
| #6 | MeSH descriptor: [Respiratory Distress Syndrome, Adult] explode all trees |
| #7 | MeSH descriptor: [Respiratory Insufficiency] explode all trees |
| #8 | MeSH descriptor: [Severe Acute Respiratory Syndrome] explode all trees |
| #9 | MeSH descriptor: [Lung Injury] explode all trees |
| #10 | respiratory distress syndrome adult |
| #11 | respiratory distress syndrome acute |
| #12 | respiratory insufficiency |
| #13 | severe acute respiratory syndrome |
| #14 | lung injury |
| #15 | #6 OR #7 OR #8 OR #9 OR #10 OR #11 OR #12 OR #13 OR #14 |
| #16 | #5 and #15 |

**Search strategy for EMBASE**

| ID | Search |
| --- | --- |
| S1 | EMB.EXACT.EXPLODE("brain natriuretic peptide") OR ab(BNP) OR ti(BNP) OR ab(B type natriuretic peptide) OR ti(B type natriuretic peptide) OR ab(brain natriuretic peptide) OR ti(brain natriuretic peptide) |
| S2 | EMB.EXACT.EXPLODE("Adult Respiratory distress syndrome") OR ab(shock lung) OR ti(shock lung) OR ab(acute respiratory distress syndrome) OR ti(acute respiratory distress syndrome) OR ab(Adult respiratory distress syndrome) OR ti(Adult respiratory distress syndrome) |
| S3 | ab(ARDS) OR ti(ARDS) OR ab(ALI) OR ti(ALI) |
| S4 | EMB.EXACT.EXPLODE("acute respiratory failure") OR ab(Respiratory Failure) OR ti(Respiratory Failure) OR ab(Respiratory Depression) OR ti(Respiratory Depression) OR ab(Ventilatory Depression) OR ti(Ventilatory Depression) OR ab(Depressions, Ventilatory) OR ti(Depressions, Ventilatory) OR ab(acute respiratory failure) OR ti(acute respiratory failure) |
| S5 | EMB.EXACT.EXPLODE("Acute lung injury") OR ab(Acute lung injuries) OR ti(Acute lung injuries) OR ab(lung injury) OR ti(lung injury) |
| S6 | ab(severe acute respiratory syndrome) OR ti(severe acute respiratory syndrome) |
| S7 | EMB.EXACT.EXPLODE("lung edema") OR ab(Cardiogenic pulmonary edema) OR ti(Cardiogenic pulmonary edema) |
| S8 | S2 OR S3 OR S4 OR S5 OR S6 OR S7 |
| S9 | S1 AND S8 |
| S10 | EMB.EXACT.EXPLODE(“animal”) NOT EMB.EXACT.EXPLODE(“human”) |
| S11 | S9 NOT S10 |
